# Risk factors for pathological fracture in patients with mandibular osteoradionecrosis

**DOI:** 10.1038/s41598-023-30735-4

**Published:** 2023-04-01

**Authors:** Hiroaki Ohori, Eiji Iwata, Daisuke Takeda, Junya Kusumoto, Takumi Hasegawa, Masaya Akashi

**Affiliations:** 1 Department of Oral and Maxillofacial Surgery, Kobe University Graduate School of Medicine, Kobe, Japan; 2 Department of Oral and Maxillofacial Surgery, Kakogawa Central City Hospital, 439 Hon-machi, Kakogawa-cho, Kakogawa, 675-8611 Japan

**Keywords:** Diseases, Risk factors, Outcomes research

## Abstract

Osteoradionecrosis (ORN) often results in pathological fractures through progression. We aimed to identify the risk factors for pathological fracture in patients with mandibular ORN. Seventy-four patients with mandibular ORN were included in this retrospective study. We investigated various risk factors for pathological fracture in patients with mandibular ORN, including number of mandibular teeth with a poor prognosis each at initial evaluation before radiation therapy (RT) and when fracture occurred, and the proportion of antibiotic administration period in a follow-up duration after RT. The rate of occurrence of pathological fractures in patients with mandibular ORN was 25.7%. The median of duration between RT completion and fracture occurrence was 74.0 months. We found that pathological fracture was significantly associated with a larger number of mandibular teeth with a poor prognosis at initial evaluation before RT (*P* = 0.024) and when fracture occurred (*P* = 0.009). Especially, a larger number of mandibular teeth with P4 periodontitis, in other words severe periodontal status, was related to pathological fracture in both timings. The proportion of antibiotic administration period in a follow-up duration was also significant risk factor (*P* = 0.002). Multivariate analyses showed statistically significant associations between pathological fracture and a larger number of mandibular teeth with a poor prognosis when fracture occurred (hazard ratio 3.669). The patient with a larger number of mandibular teeth with P4 periodontitis may have a risk of not only occurrence of ORN but resulting in pathological fracture by accumulation of infection. Surgeons should consider extraction of those teeth regardless of before/after RT if necessary for infection control.

## Introduction

Radiation therapy (RT) plays a central role in the treatment of head and neck cancer, especially in human papillomavirus (HPV)-positive oropharyngeal carcinoma^1^. Osteoradionecrosis (ORN) of the jaw is a serious and intractable adverse event that is associated with RT for head and neck cancer. Several authors reported the significant correlation between tooth extraction after RT and the occurrence of ORN^2–4^. The National Comprehensive Cancer Network (NCCN) guidelines recommend prophylactic extraction of infected teeth at least 2 weeks prior to RT^5^. The occurrence of ORN has declined with the use of Intensely Modulated Radiation Therapy (IMRT) but still ranges from 4 to 7% as reported in recent large-scale, prospective studies or as reported in a systematic review^6–11^. It has been recently found that bone exposure, but not ORN, occurs within 2 years after the completion of RT, with an incidence of 6%^12^ to 7%^13^.

ORN is defined as loss of mucosal coverage and bone exposure lasting 3–6 months^14^. When ORN develops, patients suffer from severe pain, trismus and deterioration of esthetics such as oral cutaneous fistula. This further delays the rehabilitation of patients who have completed long-term cancer treatment^15,16^. In addition, mandibular ORN often results in pathological fractures as it progresses. Patients with pathological fractures are required to undergo surgery. The most reliable treatment is resection concomitant with well-vascularized free flap transfer^17^. Surgery carries the risk of further dysfunction and esthetic disability, and the stress on patients after severe cancer treatment is immeasurable. To control infection of ORN and prevent pathological fractures of the mandible, understanding the occurrence is mandatory.

This study retrospectively investigated the risk factors for pathological fracture in patients with mandibular ORN. We focused on accumulation of infection as an important factor for resulting in pathological fracture. Therefore, we considered several risk factors which included the number of mandibular teeth with a poor prognosis at initial evaluation before RT and when fractured, and the proportion of antibiotic administration period in a follow-up duration after RT.

## Results

**Figure 1:**
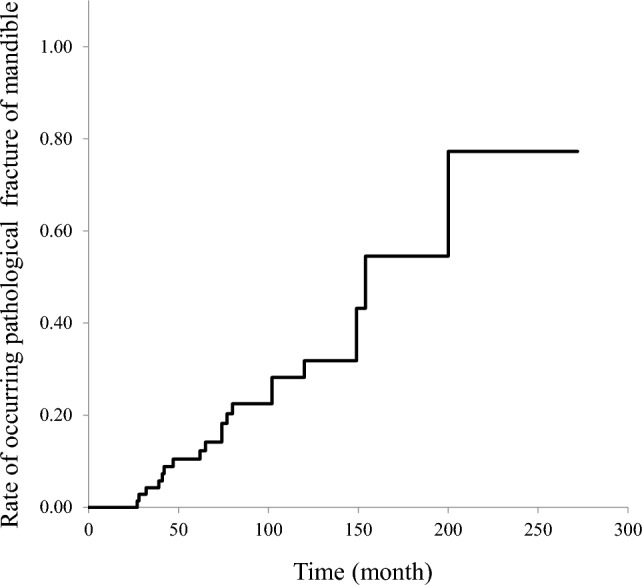
Cumulative incidence rate of pathological fracture in patients with mandibular ORN; the relationship with time after RT (months).

**Figure 2:**
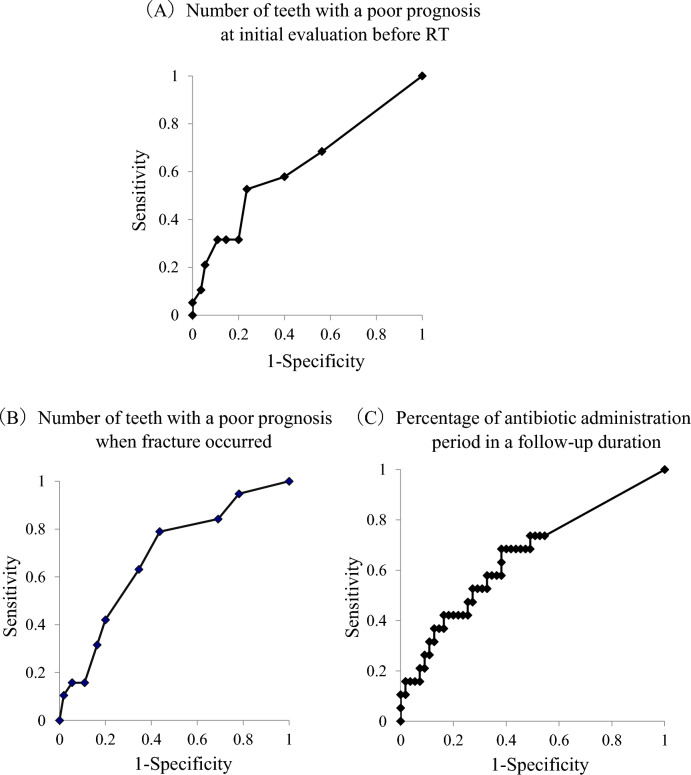
ROC curves. (**A**) The ROC curve for accuracy of number of mandibular teeth with a poor prognosis before RT. The AUC for our model was 0.625 (95% confidence interval 0.471 to 0.779). (**B**) The ROC curve for accuracy of number of mandibular teeth with a poor prognosis when fracture occurred. The AUC for our model was 0.683 (95% confidence interval 0.547 to 0.820). (**C**) The ROC curve for accuracy of proportion of antibiotic administration period in a follow-up duration. The AUC for our model was 0.655 (95% confidence interval 0.507 to 0.803).

Pathological fracture in patients with mandibular ORN occurred in 19 out of 74 patients (25.7%). The median follow-up duration after RT was 79.0 months (range, 24–272 months). The cumulative incidence rates of pathological fracture in patients with mandibular ORN at 36, 48, and 72 months after RT were 4.3%, 10.4%, and 14.1%, respectively (Fig. 1). The cutoff values associated with pathological fracture were as follows: 3.0 for the number of mandibular teeth with a poor prognosis at initial evaluation before RT (sensitivity, 52.6%; specificity, 76.4%; area under the curve [AUC], 0.625), 3.0 for the number of mandibular teeth with a poor prognosis when pathological fracture occurred (sensitivity, 79.0%; specificity, 56.4%; AUC, 0.683), and 0.004 for proportion of antibiotic administration period in a follow-up duration (sensitivity, 68.4%; specificity, 61.9%; AUC, 0.655) (Fig. 2).

**Figure 3:**
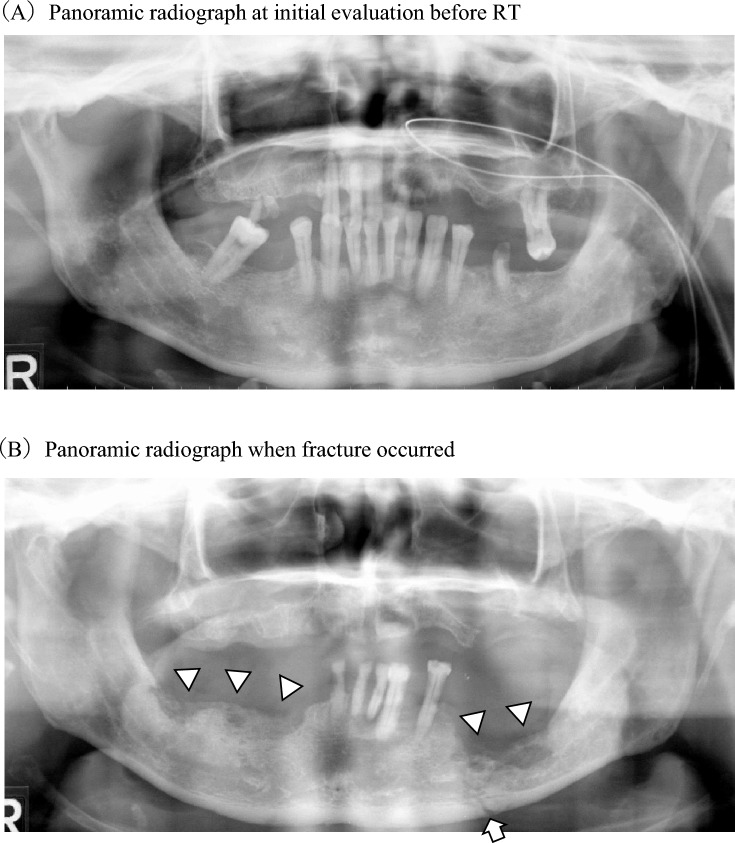
Panoramic radiograph. (**A**) Panoramic radiograph at initial evaluation before RT. (**B**) Panoramic radiograph when fracture occurred. Triangles represent the site of ORN (▽) and arrows represent the site of pathological fracture (→).

Table 1 shows the patient characteristics and results of the univariate analysis. The median age of patients with mandibular ORN was 70.0 years. The majority of sites of primary tumors were oral and oropharynx (73.0%); 17.6% of patients were treated with IMRT. Almost half of the cases occurred in the region contralateral to primary tumor (52.7%). There was significant difference between a larger number of mandibular teeth with a poor prognosis at initial evaluation before RT and pathological fracture. Especially, the larger number of mandibular teeth with P4 periodontitis among them was significant factors for occurrence of pathological fracture (*P* = 0.007). A total of 53.8% patients underwent tooth extraction before or/and after RT. If limited to P4 periodontitis, a total of 33.5% patients underwent tooth extraction before or/and after RT. In any case, the timing of tooth extraction was not associated with occurrence of pathological fracture. When pathological fracture occurred, the larger number of mandibular teeth with a poor prognosis was significant risk factor in analyses of both continuous and categorical variables. Especially, the larger number of mandibular teeth with periapical periodontitis (*P* = 0.002) and P4 periodontitis (*P* < 0.001) were significant factors for pathological fracture. There was no significant difference in the duration between RT completion and timing of fracture or most recent visit between patients with and without pathological fracture (median 74.0 vs. 81.0 months; *P* = 0.524). RT and this long period often changed oral condition of patients greatly. As a representative example, we introduce a patient with a right oropharyngeal cancer. A 56 years male had many teeth with a poor prognosis both maxilla and mandible at initial evaluation before RT (Fig. 3A). He underwent extraction of 5 out of 10 teeth with P4 periodontitis before RT and regular oral maintenance before and after RT. Unfortunately, ORN occurred in both sides of mandible, resulting in pathological fracture of left mandible 39 months after RT (Fig. 3B). When fracture occurred, remaining 5 mandibular teeth had severer periodontitis (Fig. 3B).

**Table 1:** Distribution of variables between patients with and without pathological fracture of mandible.

Variables	All patients (n = 74)	Patients with fracture (n = 19)	Patients without fracture (n = 55)	*P* value
Age (years)				
≥ 70 (median)	36 (48.6%)	8 (42.1%)	28 (50.9%)	0.599 ^c^
< 70	38 (51.4%)	11 (57.9%)	27 (49.1%)	
Sex				
Male	63 (85.1%)	15 (78.9%)	48 (87.3%)	0.458 ^c^
Female	11 (14.9%)	4 (21.1%)	7 (12.7%)	
Site of primary tumor				
Oral and oropharynx	54 (73.0%)	14 (73.7%)	40 (72.7%)	1.000 ^c^
Others	20 (27.0%)	5 (26.3%)	15 (27.3%)	
Radiation dose (Gy)				
Median years (range)	69.1 (60–81)	69.5 (60–81)	68.2 (60–80)	0.070 ^b^
IMRT				
No	61 (82.4%)	18 (94.7%)	43 (78.2%)	0.163 ^c^
Yes	13 (17.6%)	1 (5.3%)	12 (21.8%)	
With resection of mandible				
No	67 (90.5%)	17 (89.5%)	50 (90.9%)	1.000 ^c^
Yes	7 (9.5%)	2 (10.5%)	5 (9.1%)	
With neck dissection				
No	35 (47.3%)	12 (63.2%)	23 (41.8%)	0.204 ^c^
Yes	39 (52.7%)	7 (36.8%)	32 (58.2%)	
With reconstructive surgery				
No Yes	51 (68.9%) 23 (31.1%)	16 (84.2%) 3 (15.8%)	35 (63.6%) 20 (36.4%)	0.150 ^c^
With radial forearm free flap	8 (34.8%)	2 (66.6%))	6 (30.0%))	0.567 ^d^
With rectus abdominis free flap	7 (30.5%)	0 (0.0%)	7 (35.0%)	
With scapular flap	2 (8.7%)	0 (0.0%)	2 (10.0%)	
With free jejunal flap	4 (17.4%)	1 (33.4%)	3 (15.0%)	
With tongue flap	1 (4.3%)	0 (0.0%)	1 (5.0%)	
With free fibula flap	1 (4.3%)	0 (0.0%)	1 (5.0%)	
Number of mandibular teeth with a poor prognosis at initial evaluation before RT				
Median (teeth, range)	1.0 (0–12)	3.0 (0–12)	1.0 (0–10)	0.053 ^b^
≥ 3 (cut off value)	23 (31.1%)	10 (52.6%)	13 (23.6%)	0.024* ^c^
< 3	51 (68.9%)	9 (47.4%)	42 (76.4%)	
Types of teeth				
Periapical periodontitis (teeth, range)	0.0 (0–2)	0.0 (0–2)	0.0 (0–1)	0.539 ^b^
P4 periodontitis	0.0 (0–12)	2.0 (0–12)	0.0 (0–10)	0.007* ^b^
With extraction before and after RT	2 (2.7%)	0 (0.0%)	2 (3.6%)	0.770 ^d^
With extraction before RT and without extraction after RT	21 (28.4%)	6 (31.6%)	15 (27.3%)	
Without extraction before RT and with extraction after RT	1 (1.4%)	0 (0.0%)	1 (1.8%)	
Without extraction before and after RT	50 (67.5%)	13 (68.4%))	37 (67.3%)	
Untreated root remnants	0.0 (0–6)	0.0 (0–2)	0.0 (0–6)	0.247 ^b^
Root with defective prosthesis	0.0 (0–5)	0.0 (0–5)	0.0 (0–2)	0.810 ^b^
Tooth extraction				
With extraction before and after RT	11 (14.9%)	1 (5.3%)	10 (18.2%)	0.520 ^d^
With extraction before RT and without extraction after RT	21 (28.4%)	5 (26.3%)	16 (29.1%)	
Without extraction before RT and with extraction after RT	7 (9.5%)	2 (10.5%)	5 (9.1%)	
Without extraction before and after RT	35 (47.2%)	11 (57.9%)	24 (43.6%)	
Occlusal support				
Yes	50 (67.6%)	15 (78.9%)	35 (63.6%)	0.266 ^c^
No	24 (32.4%)	4 (21.0%)	20 (36.4%)	
Number of mandibular teeth with a poor prognosis when fracture occurred				
Median (teeth, range)	3.0 (0–10)	4.0 (0–10)	2.0 (0–10)	0.030* ^b^
≥ 3 (cut off value)	39 (52.7%)	15 (78.9%)	24 (43.6%)	0.009* ^c^
< 3	35 (47.3%)	4 (21.1%)	31 (56.4%)	
Types of teeth				
Periapical periodontitis (teeth, range)	0.0 (0–2)	0.0 (0–2)	0.0 (0–0)	0.002* ^b^
P4 periodontitis	1.0 (0–10)	2.0 (0–10)	0.0 (0–5)	< 0.001* ^b^
Untreated root remnants	0.0 (0–6)	0.0 (0–3)	0.0 (0–6)	0.064 ^b^
Root with defective prosthesis	0.0 (0–4)	0.0 (0–4)	0.0 (0–4)	0.994 ^b^
Location of mandibular ORN				
Ipsilateral to primary tumor	35 (47.3%)	12 (63.2%)	23 (41.8%)	0.125 ^c^
Contralateral to primary tumor	39 (52.7%)	7 (36.8%)	32 (58.2%)	
Follow-up duration after RT^a^				
Median months (range)	79.0 (24–272)	74.0 (27–200)	81.0 (24–272)	0.524 ^b^
Infection				
Yes	45 (60.8%)	14 (73.7%)	31 (56.4%)	0.276 ^c^
No	29 (39.2%)	5 (26.3%)	24 (43.6%)	
Frequency of infections				
Median (range)	1.0 (0–9)	1.0 (0–6)	1.0 (0–9)	0.190 ^b^
Proportion of antibiotic administration period in a follow-up duration				
Median (range)	0.003 (0–0.239)	0.005 (0–0.239)	0.002 (0–0.111)	0.050* ^b^
≥ 0.004 (cut off value)	33 (44.6%)	13 (68.4%)	20 (36.4%)	0.002*^c^
< 0.004	41 (55.4%)	6 (31.5%)	35 (63.6%)	

*IMRT* intensity modulated radiation therapy, *ORN* osteoradionecrosis.

*P < 0.05.

^a^Follow-up duration means duration between RT completion and the detection of pathologic fracture of mandible. In patients without pathologic fracture, the duration between RT completion and the most recent visit to our department is calculated.

^b^Mann-Whiteny U test.

^c^Fisher’s exact test.

^d^Chi-squared test.

**Figure 4:**
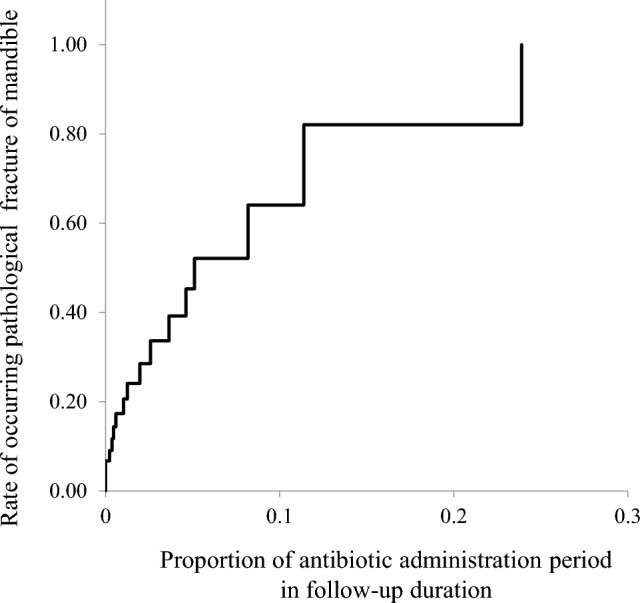
Cumulative incidence rate of pathological fracture in patients with mandibular ORN; the relationship with proportion of antibiotic administration period in a follow-up duration.

Over sixty percent of patients with mandibular ORN had an experience of infection requiring antibiotic administration. Although no significant differences were observed in the frequency of infection, the rate of infection of the patients with fracture was higher than the patients without fracture (73.7% vs 56.4%; *P* = 0.276). The proportion of antibiotic administration period in a follow-up duration was significantly associated with pathological fracture (median 0.005 vs. 0.002; *P* = 0.005). When this proportion was 5.0%, 10.0%, and 20.0%, the cumulative incidence of pathological fractures in mandibular ORN patients was 45.3%, 64.1%, and 82.0%, respectively (Fig. 4). As shown in Table 2, the associations between the variables and pathological fracture were analyzed using the Cox model. We found significant associations of pathological fracture in patients with mandibular ORN with the number of mandibular teeth with a poor prognosis when fracture occurred (hazard ratio 3.669) (Table 2).

**Table 2:** Multivariate analysis of risk factors of pathological fracture of mandible in patient with ORN.

Variables	*P* value	Hazard ratio	95% CI
Age ≥ 70 years	0.092	0.314	0.082–1.207
Sex male	0.288	2.091	0.536–8.163
Oral and oropharynx cancer	0.784	1.204	0.319–4.547
Radiation dose ≥ 70 Gy	0.211	0.37	0.078–1.757
Non-IMRT	0.977	0.967	0.093–10.096
With resection of mandible	0.142	3.83	0.639–22.974
With neck dissection	0.279	0.412	0.083–2.054
Number of mandibular teeth with a poor prognosis at initial evaluation before RT ≥ 3.0	0.272	2.008	0.578–6.977
Occlusal support	0.919	1.074	0.272–4.238
Number of mandibular teeth with a poor prognosis when fracture occurred ≥ 3.0	0.042*	3.669	1.049–12.825
Ipsilateral to primary tumor	0.792	0.854	0.264–2.763
Proportion of antibiotic administration period in a follow-up duration ≥ 0.004	0.294	1.867	0.581–5.993

*ORN* osteoradionecrosis, *CI* confidence interval.

*P < 0.05.

## Discussion

There have been numerous studies conducted on the predictive factors of occurrence of ORN^6,7,14,18^. However, clinicians who treat ORN know that not all patients with ORN have serious symptoms. When ORN worsens, aggressive surgery such as mandibular resection and reconstruction with a vascularized free flap is often necessary^16^. The most common indication for aggressive surgery is a pathological fracture. Therefore, we aimed to identify the risk factors for pathological fracture in patients with mandibular ORN. The present study included a long-term observational period (median, 79.0 months) and revealed that the incidence of pathological fracture in patients with mandibular ORN was 25.7%. The cumulative incidence rates of pathological fracture in patients with mandibular ORN at 36, 48, and 72 months after RT were 4.3%, 10.4%, and 14.1%, respectively, increased over time. Multivariate analysis showed that pathological fracture was associated with a larger number of mandibular teeth with a poor prognosis when fracture occurred.

This study focused on the role of infection in exacerbation of ORN. Infection control is an important factor in the treatment of ORN^19–21^. We hypothesized that accumulation of infection is an exacerbating factor of ORN and may be a trigger for pathological fracture. In the present study, although no significant difference in the frequency of infection was found between patients with and without fracture (median 1.0 vs. 1.0; *P* = 0.190), the rate of infection of the patients with fracture was higher (73.7% vs 56.4%; *P* = 0.276). The proportion of antibiotic administration period in a follow-up duration as an indicator of infections of ORN was significantly higher (median 0.005 vs. 0.002; *P* = 0.050). When this proportion was 5.0%, 10.0%, and 20.0%, the cumulative incidence of pathological fractures in mandibular ORN patients was 45.3%, 64.1%, and 82.0%, respectively. This proportion may be a useful indicator showing the degree of infection leading to pathological fracture. Our results indicate that the patients with fracture may suffer from longer and more severe infection after RT than those without fracture, and the accumulation of infection may cause pathological fracture. Further investigation is necessary to elucidate the role of bacterial infection in the occurrence and exacerbation of ORN.

This study also focused on the presence of teeth with a poor prognosis as one of the causes of infection of ORN. Teeth and periodontal tissue can be a route of bacterial invasion. RT causes poor circulation in periodontal tissue, and periodontal health influences the occurrence of ORN^22,23^. A recent study showed that extraction of teeth with a poor prognosis before RT significantly reduced the risk of occurrence of ORN^24^. They also showed that the presence of teeth with P4 periodontitis was significantly associated with occurrence of ORN^24^. In this study, number of mandibular teeth with a poor prognosis at initial evaluation before RT in patients with fracture was larger than patients without. It is speculated that patients who need tooth extraction at initial evaluation before RT have a poorer oral condition on a routine basis on than those who do not. Especially, a larger number of mandibular teeth with P4 periodontitis (*P* = 0.007) at the time was significantly associated with pathological fracture. The larger number of mandibular teeth with P4 periodontitis means wide range of alveolar bone loss (i.e., severe periodontal status). Interestingly, both among patients who had teeth with a poor prognosis and patients who had teeth with P4 periodontitis, the timing of tooth extraction was not associated with pathologic fractures. Koga et al. reported that the timing of tooth extraction, whether before or after RT, was not related to the occurrence of ORN^25^. However, a larger number of mandibular teeth with a poor prognosis when fracture occurred was also significantly associated with pathological fracture, especially number of mandibular teeth with periapical periodontitis (*P* = 0.002) and P4 periodontitis (*P* < 0.001). Especially, multivariate analysis showed that a larger number of mandibular teeth with a poor prognosis when fracture occurred was associated with pathological fracture. Therefore, the patient with a larger number of mandibular teeth with P4 periodontitis may have a risk of not only occurrence of ORN but resulting in pathological fracture by accumulation of infection. The prophylactic pre-RT tooth extraction of teeth with a poor prognosis, mainly teeth with P4 periodontitis, may prevent the accumulation of infection resulting in pathological fracture in patients with mandibular ORN. However, it is necessary to continue regular oral maintenance also after RT, and if the condition of the teeth becomes worse, tooth extraction should be considered each time even after RT. Although dental management is essential, especially in patients who undergo RT for head and neck cancer in whom it is so important, its criteria remain debatable^26^. A previous study reported that tooth extraction of more than eight teeth before RT significantly reduced patients’ QOL^27^. Importantly, recent clinical practice guidelines noted that dental intervention should be determined according to patients’ status and prognosis^28^. For example, in patients with a limited prognosis or elderly patients, few remaining teeth that are difficult to preserve but may be able to act as abutments for a denture will contribute to maintenance of QOL^18^. In relatively young patients with HPV-positive squamous cell carcinoma who are likely to have a good prognosis, dental management including tooth extraction should be planned from a long-term perspective.

This study has some limitations. First, although this study included many non-IMRT cases, IMRT is currently the mainstream treatment. Second, the difference in follow-up duration between IMRT and traditional RT could be a limitation. Actually, the follow-up duration of the patients underwent IMRT was significantly shorter than those underwent non-IMRT (median, 41 months vs 90 months; *P* < 0.001). However, follow-up duration alone could not be a significant risk factor for pathological fracture. Long term observation of those underwent IMRT is necessary in the future. Third, this study did not include the details of periodontal status, which surely influences ORN and the number of mandibular teeth with a poor prognosis. Finally, the treatments for mandibular ORN during the follow-up period were not unified during this study period. It remains to be elucidated whether better intervention during follow-up could prevent pathological fracture in patients with ORN. For instance, the standard of antibiotic administration may have been different from each surgeon. In the future, prospective studies are necessary to identify the risk factor for pathological fracture of mandibular ORN. In the prospective study, we should exclude patients with non-IMRT, evaluate their periodontal status, and unify the treatment method of ORN such as the standard of antibiotic administration.

In conclusion, this is the first report to have investigated the risk factors for pathological fracture in patients with mandibular ORN. The patient with a larger number of mandibular teeth with P4 periodontitis may have a risk of not only occurrence of ORN but resulting in pathological fracture by accumulation of infection. Surgeons should consider extraction of those teeth regardless of before/after RT if necessary for infection control.

## Patients and methods

### Participants

This retrospective study included patients who were diagnosed with mandibular ORN and had undergone treatment at the Department of Oral and Maxillofacial Surgery, Kobe University Hospital between April 2009 and March 2021. The patients whose following up duration was less than 24 months after RT and patients with missing data that were needed in this study were excluded. According to a previous study, ORN was defined as loss of mucosal coverage and bone exposure lasting 3–6 months^14,29^. Patients diagnosed with mandibular ORN were followed up routinely in our department. Computed tomographic imaging was performed when ORN excavation or pathological fracture was suspected during follow-up of the patients. IMRT became mainstream, replacing traditional RT, in our hospital in 2014^6^.

### Study design

**Figure 5:**
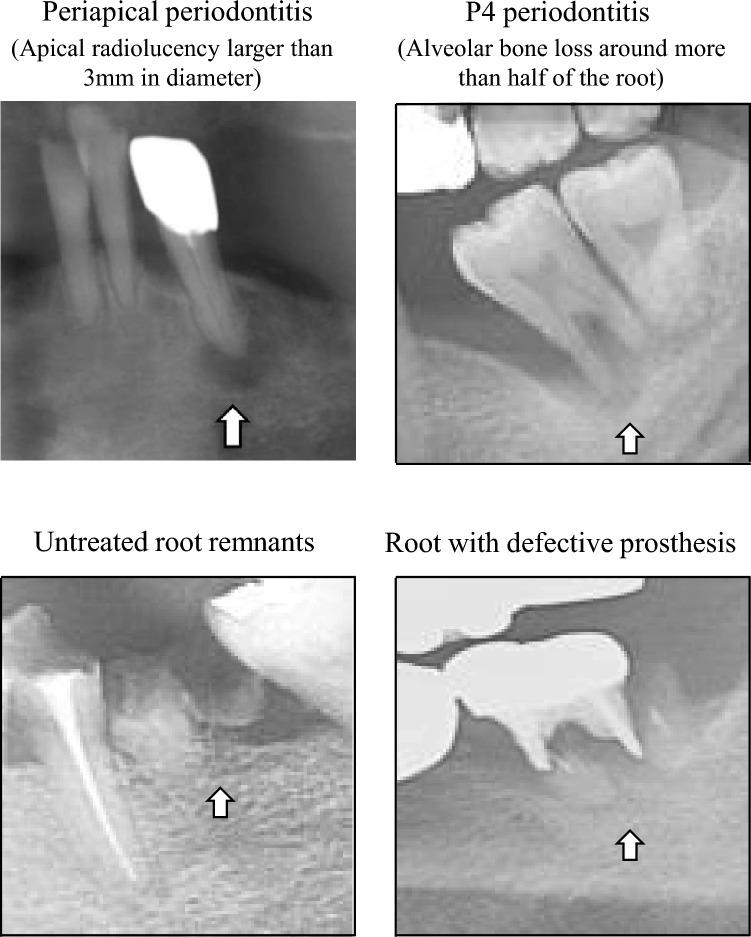
Types of teeth with a poor prognosis.

The following variables were retrospectively reviewed from the electronic medical records and investigated: (1) patient factors: age and sex; (2) factors related to the primary cancer: site of primary cancer, radiation dose, IMRT or not, with or without resection of mandible, with or without neck dissection, and with or without reconstructive surgery; (3) factors related to teeth: number of mandibular teeth with a poor prognosis each at initial evaluation before RT and when fracture occurred, types of　teeth, presence of tooth extraction, and presence of occlusal support (i.e., the existence of vertical stop); and (4) factors related to mandibular ORN: location of ORN, follow-up duration after RT (duration between RT completion and detection of pathological fracture of the mandible), presence of infection, frequency of infections, and the proportion of antibiotic administration period in a follow-up duration. We add explanations about several variables. Pathological fracture was defined as a fracture that occurred in weakened bone due to an underlying pathological effect rather than trauma^27,30^. In this study, the presence of pathological fracture was confirmed by computed tomography (CT) imaging during ORN follow-up. The teeth with a poor prognosis were defined by presence of abnormal findings on panoramic radiographs with reference to previous studies^31^. In brief, those teeth consisted of periapical periodontitis (apical radiolucency > 3 mm in diameter), P4 periodontitis (alveolar bone loss involving more than half the root), untreated root remnants, and root with defective prosthesis (Fig. 5). As a side of note, there was no tooth with root fracture. When the location of ORN infected, patients were administered antibiotics not as a treatment of ORN but for preventing spread of infection and inflammation to surrounding soft tissue caused by ORN. For the frequency of infection, the time from the start to the end of antibiotic administration was defined as a single period. The duration of antibiotic administration was counted in days regardless of route of administration (i.e., oral or intravenous administration). "The proportion of antibiotic administration period in a follow-up duration" was calculated days of antibiotic administration period divided by days of follow-up duration after RT. This calculation method is our original indicator of infection for this study. In patients without pathological fractures, the follow-up duration after RT was calculated the duration between RT completion and most recent visit to our department The number of mandibular teeth was evaluated using the most recent panoramic imaging in patients without pathological fractures.

### Statistical analysis

All statistical analyses were performed using IBM SPSS Statistics 26.0 (IBM Corp., Armonk, NY, USA) and Ekuseru-Toukei 2012 (Social Survey Research Information Co., Ltd.; Tokyo, Japan). The association of each variable with pathological fracture of the mandible was analyzed using the Mann–Whitney U test for continuous variables and Fisher’s exact test or the chi-squared test for categorical variables. *P* < 0.05 was considered statistically significant. The remaining variables were introduced into a Cox proportional hazards model. Hazard ratios and 95% confidence intervals (CIs) were calculated. As possible risk factors for pathological fracture of mandible, the number of mandibular teeth with a poor prognosis each at initial evaluation before RT and when fracture occurred, and the proportion of antibiotic administration period in a follow-up duration were evaluated by receiver-operating characteristic (ROC) curve analysis, and the cutoff values were determined. The area under the receiver operating characteristic curve measured the accuracy of discrimination (range, 0.5 to 1). The cumulative incidence of pathological fracture of the mandibular ORN was calculated using the Kaplan–Meier method.

### Ethics

The study protocol conformed to the ethical guidelines of the Declaration of Helsinki and the Ethical Guidelines for Medical and Health Research involving Human Subjects by the Ministry of Health, Labor, and Welfare of Japan. Ethical approval was obtained from the Institutional Review Board (IRB) of Kobe University Hospital (Authorization number: B210248). Japanese law does not require individual informed consent from participants in non-invasive observational trials such as the present study. Therefore, the need for informed consent was waived according to the instruction of IRB of Kobe University Hospital. As this was a retrospective study, patient identifable information was removed, and the research plan was published on the homepages of Kobe University Hospital website, along with an opt-out option in accordance with our IRB instruction.

## Data Availability

The data that support the findings of this study are available from the corresponding author upon reasonable request.
