# Author Correction: Risk factors for pathological fracture in patients with mandibular osteoradionecrosis

**DOI:** 10.1038/s41598-023-38352-x

**Published:** 2023-07-11

**Authors:** Hiroaki Ohori, Eiji Iwata, Daisuke Takeda, Junya Kusumoto, Takumi Hasegawa, Masaya Akashi

**Affiliations:** 1 Department of Oral and Maxillofacial Surgery, Kobe University Graduate School of Medicine, Kobe, Japan; 2 Department of Oral and Maxillofacial Surgery, Kakogawa Central City Hospital, 439 Hon‑machi, Kakogawa-cho, Kakogawa, 675-8611 Japan

Correction to: *Scientific Reports* 10.1038/s41598-023-30735-4, published online 01 April 2023

The original version of this Article contained an error in Table 1, where the values for “All patients (n = 74)”, “Patients with fracture (n = 19)” and “Patients without fracture (n = 55)” under the IMRT variable “Yes” and “No” were interchanged. The correct and incorrect values appear below.

Incorrect:

**Table:** 

Variables	All patients (n = 74)	Patients with fracture (n = 19)	Patients without fracture (n = 55)	*P* value
IMRT				
No	13 (17.6%)	1 (5.3%)	12 (21.8%)	0.163^c^
Yes	61 (82.4%)	18 (94.7%)	43 (78.2%)	

Correct:

**Table:** 

Variables	All patients (n = 74)	Patients with fracture (n = 19)	Patients without fracture (n = 55)	*P* value
IMRT				
No	61 (82.4%)	18 (94.7%)	43 (78.2%)	0.163^c^
Yes	13 (17.6%)	1 (5.3%)	12 (21.8%)	

The original Article has been corrected.

